# Prevalence and factors related to mouth breathing in school children at the Santo Amaro project-Recife, 2005

**DOI:** 10.1016/S1808-8694(15)30975-7

**Published:** 2015-10-19

**Authors:** Valdenice Aparecida De Menezes, Rossana Barbosa Leal, Rebecca Souza Pessoa, Ruty Mara E. Silva Pontes

**Affiliations:** a PhD, Professor; b MS. General Clinician, Dentalpediatrics; c Student; d Student. Universidade de Pernambuco - UPE

**Keywords:** dentistry pediatric, prevalence, mouth breathing

## Abstract

**Aim:**

To determine the prevalence of mouth breathing children at the santo amaro project/ esef/ upe, and study their main facial and behavior alterations.

**Study design:**

transversal study.

**Materials and methods:**

there were 150 children in the sample, with ages ranging from 8 to 10 years. Data was collected by means of a questionnaire and clinical examinations. As for their breathing assessment, two tests were carried out: test 1- breath steam against a mirror; and test 2 - water remains in the mouth with lips closed for 3 minutes.

**Results:**

mouth breathing prevalence was of 53.3%. There was no significant difference between gender, age and type of breathing. Facial alterations were: incomplete lip closure (58.8% X 5,7%), fallen eyes (40.0% X 1.4%), High palate (38.8% X 2.9%), Anterior open bite (60.0% Versus 30.0%), Hypotonic lips (3.8% X 0.0%), Circles under the eyes (97.5% Versus 77.1%).

**Conclusion:**

high mouth breathing prevalence without significant statistical difference between genders, age and type of mouth breathing. There was no association between behavior characteristics and type of breathing. There were significant differences between physical traits and breathing pattern.

## INTRODUCTION

Breathing is one of the vital functions of the human body^1^. Normal breathing should happen through the nose. However, it may be detoured to the oral via when there is some airway obstruction^2^, ^3^.

According to the literature, it is rare to have exclusive oral breathing; commonly, patients have a mixed respiratory pattern: partially oral and partially nasal^2^, ^4^, ^5^, ^6^, ^7^.

Few are the papers related to the prevalence of oral breathing in the literature, and they present percentages that vary from 5%^8^ to 75%^7^. As to gender, there is a slight predominance of this pathology in females when compared to their male counterparts^9^.

Depending on how long it lasts, oral breathing may cause functional, structural, pathological, postural, occlusal and behavioral alterations^10^, ^11^. The most common complaints of oral breathers are: breathlessness or respiratory failure, gets tired easily during physical activities, back or neck pain, olfaction and/or taste impairments, halitosis, dry mouth, wake up chocking during the night, bad sleep, day time sleepiness, dark spots underneath the eyes, sneezing, abundant saliva when speaking, among others^12^.

As physical consequences, the oral breathing child has many physical traits: long face, dropped eyes, dark spots underneath the eyes, open lips, hypotonic and dry lips, narrow nostrils, hypotonic cheek muscles, high palate, narrowing of the upper arch, and occlusal relation tending to Angle's Class II^2^, ^3^. Oral breathing also alters posture, morphology and tonicity of phonoarticulatory organs^13^.

As to behavioral alterations, we stress: restless sleep, irritability, difficulty concentration followed by a reduction in school performance and impaired sports skills, amongst others^14^, ^2^, ^5^.

Middle and long run alterations, accruing from these alterations, may have harmful consequences for the individual's quality of life due to its personal, physical, psychological and social impacts^1^, ^2^, ^15^, ^16^. Therefore, oral breathing is considered a syndrome and one of the most preoccupying public health problems today^17^.

Knowing the importance of epidemiological research today, working to reduce populational health problems, this paper aims at contributing to the study of oral breathing, through research about its prevalence and main facial and behavioral alterations associated to this respiratory pattern in school children. The participants in this study are enrolled in the Projeto Santo Amaro da Escola Superior de Educação Física (ESEF).

## MATERIALS AND METHODS

This study was carried out at the Escola Superior de Educação Física (ESEF) of the University of Pernambuco (UPE) from June to August, 2005 and is a transversal and observational type of study.

Our sample included children regularly enrolled in the Santo Amaro Project. Their ages varied between 8 and 10 years, because dental and facial alterations in the oral breather are already present at this age range^18^.

The total sample comprised 236 children; throughout a sample calculation, the necessary sample size for this study was 147 children. Thus, the number of subjects per age range was of 50 children, adding up to a total of 150.

The sample was picked by randomly selecting the students in the aforementioned age ranges. Sample inclusion criteria were: children between 8 and 10 years of age, from both genders. Sample exclusion criteria were: Children who refused to participate; Children who were not properly authorized by their parents/guardians to participate and they did not sign the Informed Consent form; children with severe respiratory disorders.

In order to collect data in order to fulfill the objectives of the present study, we used an identification form for the children, a questionnaire to identify the behavioral alterations of the oral breather, and another form about facial alterations observed at visual inspection, and the results of the tests applied aiming at completing the diagnosis of oral breathing. Clinical exams were carried out by the investigators, using PPE (personal protection equipment) and the information gathered was recorded in the standardized forms.

In order to analyze facial alterations, we checked to see whether or not the children had the following clinical signs: elongated face, dropped eyes, dark circles underneath the eyes, thin upper lip, dry lips, hypotonic lips, everted lower lip, narrow nostrils, high palate, inadequate lip sealing and anterior open bite. In order to assess some of these criteria, we were careful enough as to observe the children in their natural environment, without letting them see that they were being observed. The children who participated in the study underwent the mirror test and mouth moisture test to aid the diagnosis as follows:

Test 1: the mirror was placed underneath the child's nostrils and we checked for steam build up in the mirror face (upper or lower) due to breathing. Steam on the upper face indicated nasal breathing and on the lower or lower/upper face indicated oral breathing^2^.
Test 2: we asked the child to put some water in her/his mouth and kept the lips closed, without swallowing the water, for 3 minutes, and we observed through the lips commisure if there was any effort along the time. The children who were unable to keep their lips closed were considered oral breathers^2^, ^18^, ^35^, ^36^.

In order to consider a child as an oral breather, she/he should have at least 3 facial alterations, or steam in the lower mirror face and/or in both mirror faces, or spend less than 3 minutes with water in their mouths^2^, ^18^, ^19^, ^35^, ^36^.

For statistical analysis purposes, the children were assigned into two groups: nasal breathers and oral breathers, the latter also comprised those children with mixed breathing or exclusive oral breathers.

Calibration was carried out in four phases, intra-examiner and inter-examiner, using pictures of the children.

In order to carry out data analysis we obtained absolute and percentage distributions (Descriptive Statistics Method), and used the Pearson's Chi-Squared test (or Fisher's Exact test when there was no favorable condition to use the Chi-Squared test) and the equality in two proportions in independent group testing (inferential statistics method).

The significance level used in the statistical test was of 5% (0.05) and the intervals were obtained considering a 95.0% confidence interval. Data were plotted in the Excel spreadsheet and the software used to obtain the statistical calculations was the SAS (Statistical Analysis System) version 8.

## RESULTS

### Assessment of facial alterations by type of breathing in the whole group

Table 1 shows the list of facial alterations by type of breathing and in the whole group. From this table we stress: in the whole group the highest percentage frequencies were recorded for dark circles underneath the eyes (88.0%), anterior open bite (46.0%), inadequate lip sealing (34.0%), dropped eyes (22.0%) and high palate (22.0%).

**Table 1:** Assessment of breathing type according to facial alterations seen at visual inspection.

	Type of breathing						
Facial alterations	Nasal		Oral		Whole group		p value(2)
	N	%	n	%	n	%	
Elongated face	2	2,9	14	17,5	16	10,7	p = 0,0037*
Dropped eyes	1	1,4	32	40,0	33	22,0	p < 0,0001*
Dark spots underneath the eyes	54	77,1	78	97,5	132	88,0	p < 0,0001*
Narrow nostrils	-	-	2	2,5	2	1,3	**
Inadequate lip sealing	4	5,7	47	58,8	51	34,0	p < 0,0001*
Dry lips	-	-	5	6,3	5	3,3	**
Hypotonic lips	-	-	19	23,8	19	12,7	**
Narrow upper lip (thin)	1	1,4	11	13,8	12	8,0	p = 0,0055*
Anterior open bite	21	30,0	48	60,0	69	46,0	p = 0,0002*
High palate	2	2,9	31	38,8	33	22,0	p < 0,0001*

BASE(1)	70		80		150		

(*) – Significant difference at the level of 5.0%.

(**) – It was not possible to apply the test because of a null frequence

(1) – Considering that the same child could present more than one facial alteration, only the base is recorded in order to calculate the percentages, and not the whole.

(2) – Through the equality between two proportions test in different groups.

Among types of breathing patterns, it is possible to see that the percentages of facial alterations were correspondently higher among children who were oral breathers when compared to the nasal breathers. It may be established that the percentage differences presented in a descending order were recorded for: inadequate lip sealing (58.8% versus 5.7%), dropped eyes (40.0% x 1.4%), high palate (38.8% x 2.9%), anterior open bite (60.0% versus 30.0%), hypotonic lips (23.8% x 0.0%) and dark circles underneath the eyes (97.5% versus 77.1%).

Through the statistical analysis we see the significant difference between the two types of breathing, at the level of 5.0%, for all the items to which we could apply the comparative test (p < 0.05).

Assessment of the breathing tests results: Glatzel plate and water in the mouth

Table 2 depicts the results of the two breathing tests. In this table we can see that in the Glatzel plate steam test, most of the children (97.3%) were considered (assessed) as having upper steam formation, being 100.0% among the children with nasal breathing and 95.0% among those who were oral breathers and we did not see any significant relation between the two types of breathing (p > 0.05).

**Table 2:** Evaluation of the breathing type according to test 1 (steam on the Glatzel plate) and test 2 (time span with water in the mouth and lips sealed) results.

	Type of breathing						
Results from tests 1 and 2	Nasal		Oral		Whole group		p value
	n	%	n	%	n	%	
1 – Steam on the Glatzel metal plate face							
Upper	70	100,0	76	95,0	146	97,3	p(1) = 0,2483
Lower	-	-	3	3,7	3	2,0	
Both	-	-	1	1,3	1	0,7	

TOTAL	70	100,0	80	100,0	150	100,0	

2 – Time span with water in the mouth and lips sealed							
3 minutes	70	100,0	60	75,0	130	86,7	p(1) < 0,0001*
Less than 3 minutes	-	-	20	25,0	20	13,3	

TOTAL	70	100,0	80	100,0	150	100,0	

(*) – Significant difference at 5.0%.

(1) – Through the Pearson's Chi-Squared test.

As to the test about the time through which the child kept the water in the mouth and the lips sealed, we could see that most of them (86.7%) went up to 3 minutes, and this number was 25.0% higher among the nasal breathers investigated (100.0% versus 75.0%), and such difference reveals a significant relation between the two types of breathing, as far as test 2 is concerned (p < 0.05).

## DISCUSSION

In diagnosing a respiratory problem, it is of fundamental importance to obtain information from the parents/guardians during the medical interview. Therefore, questions about the child's sleep patterns, if he/she sleeps with the mouth opened, if there is noisy breathing, if the child lacks concentration at school, if the child feels sleepy during the day, if the pillow is wet in the morning; these questions should all be recorded, because they represent important elements in the diagnosis of oral breathing^20^.

Notwithstanding, these data alone are not enough for an accurate diagnosis of those mouth breathing individuals, and that is why it is also important to carry out the Glatzel metal plate test and the time through which the child keeps water in her/his mouth with the lips sealed and without swallowing it, since we have seen that the results differ and complete each other.

As to the prevalence of oral breathing, despite the few studies that have been carried out with this purpose, we have seen disagreements in the literature. In the present study we noticed that most of the children (53.3%) were considered oral breathers. The highest percentages of this alteration were seen in a study about oral suction habits in a low income population, which was of 77.78%^7^ in prevalence. Other epidemiological surveys have reported on percentages that varied between 4.5% and 34%^8^, ^21^, ^22^, ^24^, ^26^, ^28^.

These percentage differences may be justified by the diagnostic criteria and the different methodologies used in the studies. In this investigation, we did not record exclusive oral breathers, gathering both the exclusive oral breathers and the mixed breathing children in the same group, as advocated in the literature. However, some studies did not specify the criteria adopted in details.

According to many authors^2^, ^4^, ^5^, ^6^, ^7^, ^27^ it is rare to find an exclusively oral breathing pattern, and what is more common is for the patient, for some factor that makes it difficult for him/her to breathe freely through the nose (allergies, adenoid, hypertrophied tonsils, tumors, sinusitis, rhinitis, etc) to carry out a mixed type of breathing, partially oral and partially nasal. Within this context, some authors believe that the use of the term oral breather is somewhat improper and it should be replaced by the term: insufficient nasal breather. We also agree as to the inadequate use of the term “oral breather” since in our study and in the others hereby mentioned, exclusive oral breathing is rare or inexistent.

As to gender, we noticed that despite a higher percentage seen in males (53.75%) when compared to females (46.25%), this difference was not statistically significant among oral breathers, and such data corroborate other studies in which there was a slight difference in the variable being analyzed8. On the other hand, other investigations reported that there is a slight predominance of this pathology in females when compared to males^9^. However, these data do not seem to be relevant, since this gender predominance is mild, seen both in our study as in others mentioned above.

As to facial alterations that affect oral breathers, the highest percentages seen in the present study were: anterior open bite (60%), inadequate lip sealing (58.8%) and high palate (38.8%). These results are in agreement with those from a study in which the main craniofacial alterations seen in oral breathers were: open bite, high palate, malocclusion^28^. In a retrospective study, it was also possible to notice that most oral breathers also had malocclusion, and the anterior open bite was the most frequent type found^29^.

Other alterations such as dark spots underneath the eyes (97.5%) and dropped eyes (40%) which represented high percentages among the population studied were also mentioned by other authors as being facial alterations commonly found in patients with oral breathing syndrome^2^, ^11^.

Among oral breathers and nasal breathers, respectively, the percentage differences seen in descending order were: inadequate lip sealing (58.8% versus 5.7%), dropped eyes (40.0% x 1.4%), high palate (38.8% x 2.9%), anterior open bite (60.0% versus 30.0%), hypotonic lips (23.8% x 0.0%) and dark spots underneath the eyes (97.5% versus 77.1%).

In the long run, individuals who have trouble breathing may develop a number of disorders, such as: craniofacial alterations (long and narrow face), malocclusion, high palate, hypotonic lips and tongue, dry lips, sleepy face, deep dark spots underneath the eyes, greater likelihood of developing dental cavities, speech disorders, postural and gait alterations, all which interfere in school and work performance, and also in social relationships^2^, ^5^, ^30^, ^31^, ^32^, ^33^. Oral breathers are more prone to having repeated flue episodes, spasmodic cough and hoarseness. Moreover, they develop facial deformations called “facies adenoid”^20^.

Analyzing the data from the present study, it was also possible to see that of the facial alterations seen, percentages were correspondingly higher in oral breathing children than in nasal breathers, with a significant association, in agreement with the results found by aforementioned authors, despite the fact that these data support the statement that not all individuals with oral breathing patterns present these characteristics^34^.

Notwithstanding, today, oral breathing is one of the most concerning problems for public heath^12^, ^17^. Depending on its duration, it may cause a number of alteratins^1^, ^10^, ^11^, ^16^. These alterations may bring about harmful consequences to the individual's quality of life due to its personal, physical, psychological and social impact. Therefore, its treatment should be multidisciplinary, involving early prevention and treatment strategies in order to avoid symptomatic treatments^2^, ^3^, ^11^.

Considering the high prevalence of oral breathing in the population studied, we submit that health policies should be implemented in order to improve the life quality of oral breathing children, and we stress the need for new studies with a larger number of children.

## CONCLUSIONS

1. The prevalence of oral breathing was high, without significant difference between genders.
2. There were no relations between breathing pattern and behavioral alterations.
3. Physical characteristics related to oral breathing were: elongated face, dropped eyes, dark spots underneath the eyes, narrow nostrils, inadequate lip sealing, dry and hypotonic lips, narrow upper lip (thin), anterior open bite and high palate.
